# Ultralow Lattice Thermal Conductivity and Large Glass‐Like Contribution in Cs_3_Bi_2_I_6_Cl_3_: Rattling Atoms and *p*‐Band Electrons Driven Dynamic Rotation

**DOI:** 10.1002/advs.202406380

**Published:** 2024-09-18

**Authors:** Yu Wu, Jialin Ji, Yimin Ding, Jiong Yang, Liujiang Zhou

**Affiliations:** ^1^ Yangtze Delta Region Institute (Huzhou) University of Electronic Science and Technology Huzhou Zhejiang 313001 China; ^2^ College of Biological Chemical Sciences and Engineering Jiaxing University Jiaxing Zhejiang 314001 China; ^3^ Materials Genome Institute Shanghai University Shanghai 200444 China; ^4^ Zhejiang Laboratory Hangzhou Zhejiang 311100 China; ^5^ School of Physics, State Key Laboratory of Electronic Thin Films and Integrated Devices University of Electronic Science and Technology Chengdu Sichuan 610054 China

**Keywords:** DFT calculations, halide perovskites, thermal conductivity

## Abstract

Understanding the origin of ultralow lattice thermal conductivity κ_*L*_ of halide perovskites is of great significance in the energy conversion field. The soft phonon modes and the large anharmonicity corresponding to the dynamic rotation of halogen atoms play an important role in limiting the thermal transport of halide perovskites. The dynamic rotation has long been thought to originate from the electrostatic repulsion of lone pairs around halogen atoms. Here, by studying the layered perovskite Cs_3_Bi_2_I_6_Cl_3_, it is found that the interaction between the lone pairs contributed by the *s* bands of halogen atoms is short‐range, and the dynamic rotation is really driven by the occupied *p*‐band electrons. It dominates Cs_3_Bi_2_I_6_Cl_3_ with ultralow κ_*L*_, < 0.2 W mK^−1^ at 300 K. Moreover, soft optical phonons are presented ≈1 and 2.2 THz that constitute relatively flat and dense bands due to the rattling Cs and Cl atoms, contributing a large glass‐like component to the κ_*L*_. The results have important implications for understanding the origin of the ultralow κ_*L*_ in halide perovskites and for designing novel perovskites to serve the energy conversion field.

## Graphical abstract

This work reveals that the *p*‐band electrons of halogen atoms in Cs_3_Bi_2_I_6_Cl_3_ perovskite material can effectively drive the dynamic rotation mode. The resulting soft acoustic phonons and large lattice anharmonicity contribute to ultralow lattice thermal conductivity. In addition, the dense phonon spectral features contributed by the rattling atoms bring about a large glass‐like component in the thermal conductivity.

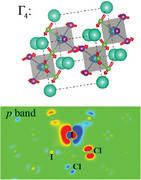

## 1 Introduction

Ultralow thermal conductivity materials are of great significance for a variety of applications, such as thermoelectrics,^[^ ^1^ ^]^ photovoltaics,^[^ ^2^ ^]^ and thermal insulation.^[^ ^3^ ^]^ Defect engineering,^[^ ^4^ ^]^ nanostructural modifications,^[^ ^5^ ^]^ and interface engineering^[^ ^6^ ^]^ strategies have been widely used to reduce lattice thermal conductivity κ_*L*_ by introducing additional phonon scattering. However, these strategies can also interfere with electron transport while reducing the κ_*L*_. Therefore, it is of great value to search for materials with intrinsically low κ_*L*_. It has been demonstrated that the soft phonon modes and large lattice anharmonicity can induce low κ_*L*_ in materials. The former can result in low phonon group velocities and large phonon scattering phase space. The latter is associated with large phonon scattering strength. Heavy elements,^[^ ^7^ ^]^ reduction of lattice symmetry,^[^ ^8^ ^]^ lone‐pair electrons,^[^ ^9^, ^10^, ^11^ ^]^ rattling atoms,^[^ ^12^, ^13^, ^14^ ^]^ anti‐bonding valence bands,^[^ ^15^, ^16^ ^]^ and resonant bonding^[^ ^17^, ^18^, ^19^, ^20^ ^]^ have been found to trigger soft phonon modes and strong anharmonicity in compounds.

All‐inorganic halide perovskites are rapidly emerging as promising alternatives to organic‐inorganic perovskites because of their superior stabilities and comparable properties, such as strong emission,^[^ ^21^ ^]^ high fluorescence quantum yield^[^ ^22^ ^]^ and tunable bandgap covering the entire visible spectrum.^[^ ^22^ ^]^ In addition, all‐inorganic halide perovskites usually exhibit intrinsically ultralow κ_*L*_ and have potential applications in the field of thermoelectrics.^[^ ^23^ ^]^ Based on the generalized view, the ultralow κ_*L*_ of all‐inorganic halide perovskites is mainly related to the following two microscopic mechanisms: 1) Rattling motion of metal cations due to weak chemical bonding.^[^ ^24^, ^25^, ^26^ ^]^ This results in soft phonon modes and enhanced phonon scattering channels due to avoid‐crossing phenomena in phonon dispersion. 2) Dynamic rotation of halogen atoms in anion groups, which form a “cluster” with heavy mass and bring about large lattice anharmonicity.^[^ ^24^, ^25^, ^27^ ^]^ However, the following outstanding questions exist regarding the thermal transport mechanism of halide chalcogenides: 1) The κ_L_ of the CsPbI_3_ and PbI_6_ frameworks was compared and it was found that the rattling effect of Cs atoms brought about an increase in the κ_L_ of the system instead of a decrease.^[^ ^28^ ^]^ 2) The origin of the dynamic rotation is controversial. It is generally thought to originate from the electrostatic repulsion of lone pairs with *s*‐band electrons,^[^ ^24^, ^25^, ^29^ ^]^ but Lee et al., reported that the interaction of *s*‐band electrons is short‐range.^[^ ^17^ ^]^ Therefore, exploring the mechanism of rattling atoms and the origin of dynamic rotation is critical to understanding the thermal transport properties of halide perovskites.

Here, we have systematically investigated the lattice thermal transport properties of Cs_3_Bi_2_I_6_Cl_3_ by first‐principles simulations. As a layered halide perovskite, Cs_3_Bi_2_I_6_Cl_3_ has recently been synthesized and exhibits ultralow κ_*L*_ (≈0.22 W mK^−1^ at 300 K) with glass‐like temperature dependence.^[^ ^30^ ^]^ Our theoretical results are in good agreement with experiments. The dynamic rotation formed by Cl and I atoms mainly affects the particle‐like component in κ_*L*_, which is the root cause of ultralow κ_*L*_. The dynamic rotation causes the softening of acoustic and low‐frequency optical phonons, which increases acoustic–acoustic–optical (aao) scattering channels. At the same time, it also brings about large lattice anharmonicity. Importantly, we show that the dynamic rotation originates from the electrostatic repulsion of the occupied *p*‐band electrons rather than the lone pairs. The large glass‐like component in κ_*L*_ comes from the flat and dense phonon dispersion features formed by rattling Cs and Cl atoms in Cs_3_Bi_2_I_6_Cl_3_. Our results reveal the source of dynamic rotation and large glass‐like thermal transport in halide perovskite Cs_3_Bi_2_I_6_Cl_3_, which is of guiding significance for designing new perovskites with low thermal conductivity.

## 2 Results and Discussion

**Figure 1:**
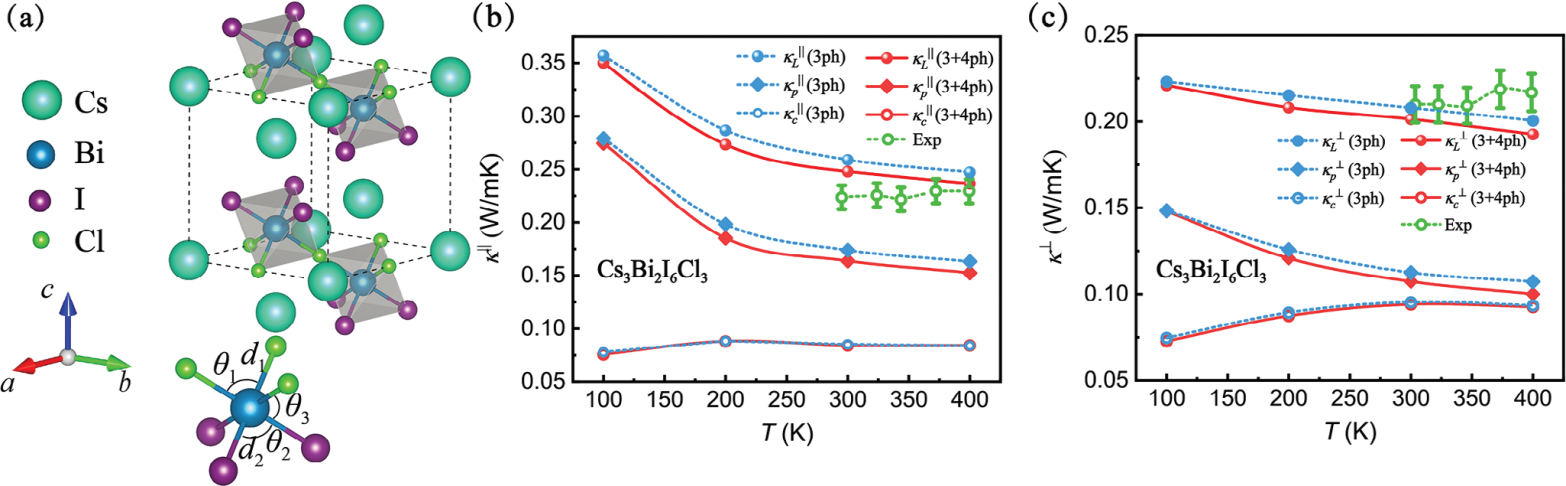
a) Crystal structures of Cs_3_Bi_2_I_6_Cl_3_. Calculated temperature‐dependent κ_L_ of Cs_3_Bi_2_I_6_Cl_3_ along b) in‐plane and c) cross‐plane directions. Experimental values^[^ ^30^ ^]^ are shown in green circles for comparison.

In experiment, Cs_3_Bi_2_I_6_Cl_3_ has a 2D layered perovskite structure with a space group *P*‐3*m*1,^[^ ^30^ ^]^ which is isostructural to Cs_3_Bi_2_Br_9_ and α‐Cs_3_Sb_2_I_9_. The configurations of Cs_3_Bi_2_I_6_Cl_3_ are shown in **Figure** **1**a. For Cs_3_Bi_2_I_6_Cl_3_, each BiCl_3_I_3_ octahedron connects the adjacent octahedron by Cl atoms in one layer and the I atoms are located at the boundaries of different layers. The optimized lattice parameters for Cs_3_Bi_2_I_6_Cl_3_ are *a* = *b* = 8.20 Åand *c* = 9.94 Å, which agree well with the experimental value (*a* = *b* = 8.24 Å and *c* = 10.03 Å).^[^ ^30^ ^]^

There is a local structural distortion in the octahedra of Cs_3_Bi_2_I_6_Cl_3_. Two bond lengths exist between the center Bi atom and the ligand halogen atoms, *d*_1_(Bi–Cl) = 2.87 Åand *d*_2_(Bi–I) = 2.94 Å. Besides, there are three bond angles between the center Bi atom and the ligand halogen atoms, θ_1_(Cl–Bi–Cl) = 91.13°, θ_2_(I–Bi–I) = 91.49° and θ_3_(Cl‐Bi‐I) = 88.69°. The octahedral distortion parameters α and β are calculated to evaluate the degree of octahedral distortion in Cs_3_Bi_2_I_6_Cl_3_, which are defined as $\alpha =\frac{1}{6}\sum_{i=1}^{6}{\left(\frac{d_{i}-d}{d}\right)}^{2}$ and $\beta =\sum_{i=1}^{12}\left|\theta_{i}-90\right|$. In the formula, *d*_*i*_ is the distance between the center Bi atom and the ligand atom *i*, *d* is the average value of *d*_*i*_, and θ_*i*_ is the angle between the center Bi atom and the ligand atoms. The α and β are calculated to be 1.45 × 10^−4^ and 15.71, respectively. The distorted structures are usually accompanied by large lattice anharmonicity.^[^ ^31^, ^32^, ^33^ ^]^

Previous experimental results have shown that κ_*L*_ of Cs_3_Bi_2_I_6_Cl_3_ exhibits an anomalous glass‐like temperature dependence.^[^ ^30^ ^]^ Therefore, it is essential to evaluate the thermal transport properties of Cs_3_Bi_2_I_6_Cl_3_ including both the particle‐like and glass‐like contributions. In the framework of the Wigner formalism,^[^ ^34^, ^35^ ^]^ κ_*L*_ can be expressed as

(1) $$\kappa_{L}^{\alpha \beta }=\kappa_{p}^{\alpha \beta }+\kappa_{c}^{\alpha \beta }$$

where $\kappa_{p}^{\alpha \beta }$ is related to the particle‐like propagation of phonon wavepackets discussed by Peierls's semiclassical picture, written as

(2) $$\kappa_{p}^{\alpha \beta }=\frac{1}{VN}\sum_{qs}C_{q}^{s}v_{q,\alpha }^{s}v_{q,\beta }^{s}\tau_{q}^{s}$$

$\kappa_{c}^{\alpha \beta }$ is the glass‐like component associated with the wave‐like tunneling and loss of coherence between different phonon branches *s* and *s*′, written as

(3) $$\begin{array}{cc} \kappa_{c}^{\alpha \beta }= & \frac{\hbar^{2}}{k_{B}T^{2}VN}\sum_{q}\sum_{s\neq s^{\prime }}\frac{\omega_{q}^{s}+\omega_{q}^{s^{\prime }}}{2}v_{q,\alpha }^{s,s^{\prime }}v_{q,\beta }^{s,s^{\prime }}\\ & \times \frac{\omega_{q}^{s}n_{q}^{s}\left(n_{q}^{s}+1\right)+\omega_{q}^{s^{\prime }}n_{q}^{s^{\prime }}\left(n_{q}^{s^{\prime }}+1\right)}{4{\left(\omega_{q}^{s}-\omega_{q}^{s^{\prime }}\right)}^{2}+{\left(\Gamma_{q}^{s}+\Gamma_{q}^{s^{\prime }}\right)}^{2}}\times \left(\Gamma_{q}^{s}+\Gamma_{q}^{s^{\prime }}\right) \end{array}$$

In these expressions, α and β are the Cartesian indices, *V* is the volume of the unit cell, *N* is the number of sampled phonons in the first Brillouin zone. $C_{q}^{s}$, $v_{q}^{s}$, $\tau_{q}^{s}$, $\omega_{q}^{s}$, $v_{q}^{s,s^{\prime }}$, $\Gamma_{q}^{s}$ are the heat capacity, the group velocity, the lifetime, the phonon frequency, the interband velocity matrix element and the scattering rate of a phonon mode with wavevector q. $n_{q}^{s}$ is the equilibrium Bose–Einstein distribution.

Figure 1b,c shows the obtained temperature–dependent lattice thermal conductivity along in‐plane (||) and cross‐plane (⊥) directions, respectively. $\kappa_{p}^{\left|\right|}$ ($\kappa_{p}^{⊥}$) is 0.18 W mK^−1^ (0.11 W mK^−1^) at 300 K considering 3ph scattering and decreases to 0.16 W mK^−1^ (0.10 W mK^−1^) when involving 4ph scattering. κ_*c*_ is insensitive to 4ph scattering and has weak anisotropy. Considering 3ph and 4ph scattering, $\kappa_{c}^{\left|\right|}$ ($\kappa_{c}^{⊥}$) is calculated to be 0.08 W mK^−1^ (0.09 W mK^−1^) at 300 K. Due to the considerable and almost isotropic κ_*c*_, the κ_*L*_ exhibits weak anisotropy with $\kappa_{L}^{\left|\right|}/\kappa_{L}^{⊥}$ ratio of 1.27 which is close to the experimental value of 1.1.^[^ ^30^ ^]^ Moreover, $\kappa_{p}^{⊥}$ and $\kappa_{c}^{⊥}$ show obvious opposite trends with temperature, leading to a weak temperature dependence of $\kappa_{L}^{⊥}$ (∝*T* ^−0.09^). The experimental κ_*L*_ maintains a continuous upward trend at 0–100 K and gradually becomes stable with the further rise in temperature,^[^ ^30^ ^]^ which is different from our theoretical results. This is due to the dominance of boundary and defect scattering by phonons at low temperatures,^[^ ^7^ ^]^ which is not taken into account in our calculations.

**Figure 2:**
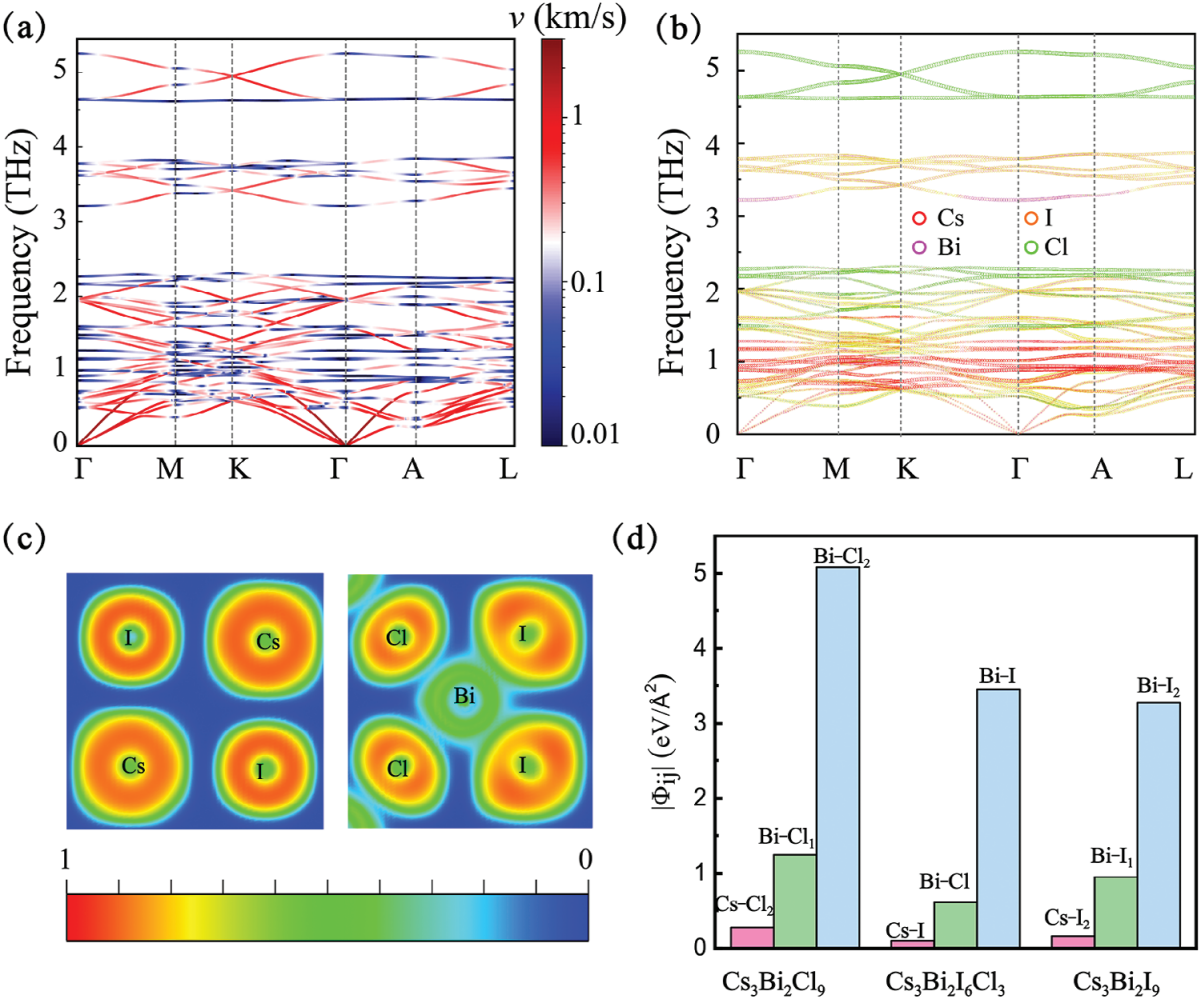
a) Phonon dispersion of Cs_3_Bi_2_I_6_Cl_3_ at 300 K with group velocities projection. b) The projected phonon dispersion of Cs_3_Bi_2_I_6_Cl_3_ weighted by their constituted atoms. c) Electronic localization function (ELF) of Cs_3_Bi_2_I_6_Cl_3_. d) Second‐order IFCs for the nearest neighboring atomic pairs in Cs_3_Bi_2_Cl_9_, Cs_3_Bi_2_I_6_Cl_3_, and Cs_3_Bi_2_I_9_ with the same crystal structure.

The phonon dispersion of Cs_3_Bi_2_I_6_Cl_3_ at 300 K with group velocities projection is shown in **Figure** **2**a. The phonon dispersion is flat in most regions and phonons have ultralow group velocities of less than 2 km s^−1^. The phonon dispersion ≈1 and 2.2 THz is almost horizontal with phonon group velocities of ≈0.1 km s^−1^. Low group velocities will directly result in low κ_*p*_ according to Equation (2). On the other hand, the ultralow group velocities are usually accompanied by soft phonon modes, which lead to a dense distribution of phonon spectral lines. In this case, the frequency difference of phonon pairs ($\omega^{s}-\omega^{s^{\prime }}$) will be reduced to improve the glass‐like contribution to κ_*L*_. Moreover, the low‐frequency optical phonon modes in the range of 0.5–1 THz overlap with the acoustic modes, which implies large scattering channels between acoustic and optical modes. Figure 2b shows the projection of the phonon eigenvectors of each atom onto the phonon dispersion. The phonon dispersion near 1 and 2.2 THz is mainly contributed by Cs and Cl atoms, respectively. The phonon group velocities are closely related to the chemical bonding. Low phonon group velocities suggest a weak bonding environment in the material.

The bonding environment can be reflected by the electron localization function (ELF), as shown in Figure 2c. The ELF is ≈0.5 between Bi and I atoms, indicating that Bi and I atoms are connected by covalent bonding in BiCl_3_I_3_ octahedral structures. The ELF near Cs atoms is almost equal to 0, which implies a weak electrostatic interaction between Cs atoms and BiCl_3_I_3_ structures. Notably, the ELF between Bi and Cl atoms is much smaller than that between Bi and I atoms, suggesting that their weaker bonding in BiCl_3_I_3_ structures. The weak interaction of Cs and Cl atoms with surrounding atoms can be further verified by the magnitude of the second‐order interaction force constants (IFCs). As shown in Figure 2d, the IFC between Bi–I pair (3.45 eV Å^−2^) is ≈6 and 30 times greater than that between Bi–Cl and Cs–I pairs in Cs_3_Bi_2_I_6_Cl_3_. Due to weak bonding features, Cs and Cl atoms can be seen as rattling atoms with large‐amplitude random movements in Cs_3_Bi_2_I_6_Cl_3_.^[^ ^29^ ^]^ The huge difference in IFCs between Bi–Cl and Bi–I pairs is not caused by atomic species. Figure 2d also shows the IFCs in Cs_3_Bi_2_Cl_9_ and Cs_3_Bi_2_I_9_ obtained by element substitution from Cs_3_Bi_2_I_6_Cl_3_. The IFCs also vary greatly between Bi–Cl_1_ (Bi–I_1_) and Bi–Cl_2_ (Bi–I_2_) pairs in Cs_3_Bi_2_Cl_9_ (Cs_3_Bi_2_I_9_). Therefore, the difference of IFCs between Bi–Cl and Bi–I pairs in Cs_3_Bi_2_I_6_Cl_3_ is determined by structural features. Each Cl atom is shared by two neighboring octahedra and the electrons obtained during the bonding process are divided equally between two Bi–Cl bonds, making them less strong than Bi–I bonds.

**Figure 3:**
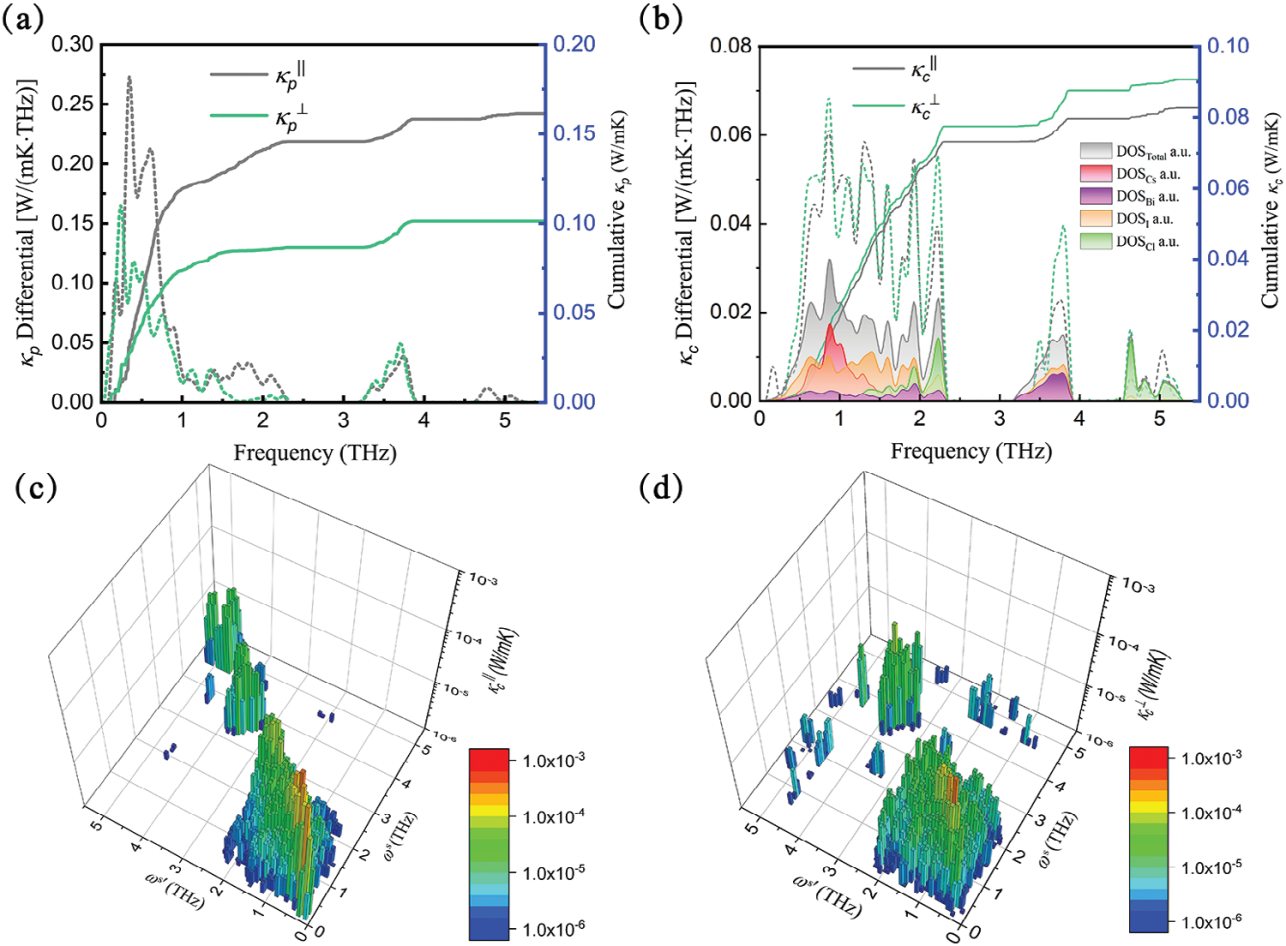
Calculated cumulative and differential a) κ_*p*_ and b) κ_*c*_ as a function of phonon frequencies for Cs_3_Bi_2_I_6_Cl_3_ at 300 K. The resolved κ_*c*_ associated with various pairs of phonon frequencies (ω^*s*^ and $\omega^{s^{\prime }}$) along (c) in‐plane and d) cross‐plane directions.

To further investigate which part of phonons has a major contribution to κ_*p*_ in Cs_3_Bi_2_I_6_Cl_3_, **Figure** **3**a shows the frequency‐dependent cumulative κ_*p*_ and its differential at 300 K. The most significant increase in cumulative κ_*p*_ is observed in the frequency ranges of 0–1 THz consisting mainly of acoustic phonons with relatively high group velocities. In addition, optical phonons in the 1–2.5 and 3–4 THz make a small contribution to κ_*p*_. The cumulative κ_*c*_ and its differential at 300 K are shown in Figure 3b. The phonons contributing to κ_*c*_ have a wide frequency range and are mainly concentrated in 0.5–2.4 THz. The atom‐resolved density of states (DOS) of phonons is also shown in Figure 3b. The curve profiles of DOS and cumulative κ_*c*_ differential are almost identical. This is because the region of higher DOS has more phonon pairs with similar frequencies which contribute mainly to κ_*c*_. The I atoms contribute to the DOS and cumulative κ_*c*_ differential at almost all frequencies, due to the delocalized covalent bonding between Bi–I atoms.^[^ ^29^ ^]^ Two significant peaks ≈1 and 2.2 THz appear on DOS and cumulative κ_*c*_ differential, contributed by Cs and Cl atoms, respectively. Combined with the analysis of Figure 2d, it is clear that the weak interaction of Cl and Cs atoms with the surrounding environment plays an important role in glass‐like thermal transport. In Figure 3c,d, we depict the magnitude of κ_*c*_ component resolved by frequencies of various phonon pairs at 300 K. It is verified that the dense quasi degenerate ($\omega^{s}∼\omega^{s^{\prime }}$) phonon pairs contribute most to κ_*c*_.

**Figure 4:**
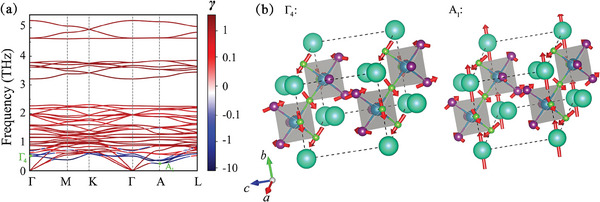
a) Phonon dispersion of Cs_3_Bi_2_I_6_Cl_3_ at 300 K with Grüneisen parameters projection. b) Atomic vibrations at Γ_4_ and A_1_ points in the phonon dispersion.

To understand the anharmonicity in Cs_3_Bi_2_I_6_Cl_3_, **Figure** **4**a shows the mode‐resolved Grüneisen parameters γ. Most acoustic phonons and low‐frequency optical phonons near 0.5 THz have large negative γ values. The phonons in these regions contribute mainly to κ_*p*_, which is inversely proportional to the square of γ.^[^ ^36^ ^]^ We have visualized the eigenvectors of two phonon modes with considerable negative γ (the 4th band at Γ point Γ_4_ and 1st band at A point $A_{1}$) in Figure 4b. Both Γ_4_ and $A_{1}$ modes involve the rotations of Bi_2_I_3_Cl_3_ octahedra with the displacement of Cl and I atoms. During the rotations, there is no significant change in the relative positions of the Cl and I atoms. Thus, the Cl and I atoms form a cluster during vibration. The cluster possesses a larger “atomic” mass and induces soft phonon modes which typically exhibit strong anharmonicity.^[^ ^25^, ^27^ ^]^

**Figure 5:**
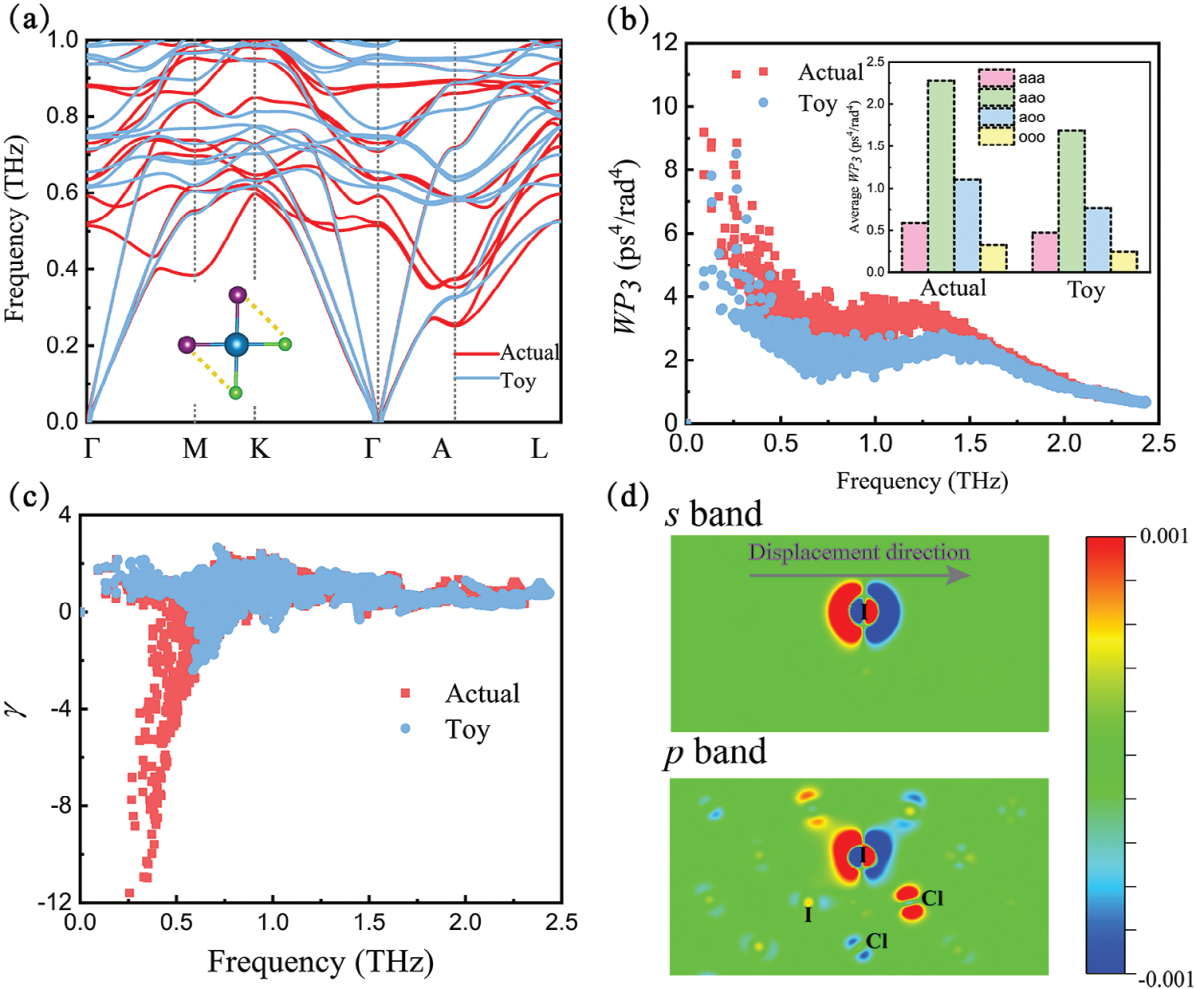
a) Actual phonon dispersion and toy model phonon dispersion by reducing the strength of the second‐order IFCs of the neighboring Cl‐I pairs at 300 K. b)The weighted 3ph phase space (*WP*_3_) c) Grüneisen parameters γ of actual and toy model in Cs_3_Bi_2_I_6_Cl_3_. The inset shows the channel‐resolved average *WP*_3_. d) Absolute charge density changes due to the displacement (0.1 Åalong the direction shown in the figure) of the I atom within a 2 × 2 × 1 supercell.

To clarify the origin of dynamic rotation and its influence on the lattice thermal transport properties, we artificially reduce the strength of the second‐order IFCs of different atomic pairs by toy model in Cs_3_Bi_2_I_6_Cl_3_ and find that the interaction between atoms in the neighboring vertices of BiCl_3_I_3_ octahedra is the inducement of the formation of the dynamic rotation. There are two steps for establishing new IFCs by toy model in this work^[^ ^27^, ^37^ ^]^: 1) the Φ_*ij*, *j* ≠ *i*_ (toy model) reduced to $\frac{1}{2}\Phi_{ij,j\neq i}$ (actual model); 2) the Φ_*ii*_ (toy model) reconstructed by calculating −∑Φ_*ij*, *j* ≠ *i*_ (toy model) for satisfying the acoustic sum rule. **Figure** **5**b shows the phonon dispersion of Cs_3_Bi_2_I_6_Cl_3_ when reducing the strength of the IFCs of the nearest neighboring of Cl–I pairs to half by toy model. Compared with the actual phonon dispersion, the acoustic phonons and low‐frequency optical phonons show significant hardening in the toy model phonon dispersion. In this process, the group velocities of acoustic phonons increase. Besides, the frequency difference of the acoustic and optical phonons increases, which will weaken the coupling between them. Figure 5b shows the weighted 3ph phase space (*WP*_3_) of actual and toy models in Cs_3_Bi_2_I_6_Cl_3_ at 300 K. The actual *WP*_3_ is much larger than that in toy model between 0 and 1.5 THz which covers the phonons making the main contribution to κ_*p*_. The inset of Figure 5b shows the channel‐resolved average *WP*_3_. It can be seen that the acoustic–acoustic–optical (aao) processes dominate the scattering of acoustic phonons which decrease significantly in the toy model. Figure 5c shows frequency‐dependent γ in actual and toy models. In the region of acoustic phonons and low‐frequency optical phonons, the magnitude of γ drops sharply in the toy model. The phonon dispersion after reducing the IFCs of the nearest Cl–Cl and I–I pairs is shown in Figure S1a,b (Supporting Information), respectively, exhibiting weak effects compared to the Cl–I pairs.

The dynamic rotation in perovskites has long been regarded to be caused by the electrostatic repulsion of lone pairs around the anionic species. For example, Biswas et al., reported the rotational vibration of Cl atoms induced by 3s^2^ lone pairs in 2D perovskite Cs_2_PbI_2_Cl_2_.^[^ ^25^ ^]^ The halogen atoms in Cs_3_Bi_2_I_6_Cl_3_ has very weak *sp*‐hybridization as seen in Figure S2 (Supporting Information) and we plot the change in the distribution of the *s*‐band and *p*‐band electrons separated according to electron energy^[^ ^17^ ^]^ with the displacement of the I atom along the rotational direction in Figure 5d. Our results show that the interaction between the lone pairs contributed by the *s*‐band electrons is short‐range and the interactions of neighboring Cl–I pairs are formed by the occupied *p*‐electrons.

**Figure 6:**
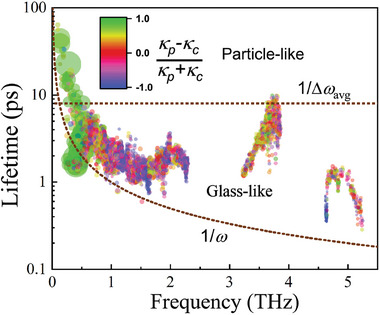
Distribution of phonon lifetimes as a function of phonon frequencies. The area of each scatter point is proportional to the contribution to the κ_*L*_ and colored according to the origin of the contribution, where particle‐like is green (c = 1), glass‐like is blue (c = ‐1). The Ioffe–Regel (τ_*Ioffe*−*Regel*_ = 1/ω) and Wigner (τ_*Wigner*_ = 1/Δω_*avg*_) limits are represented by dotted brown lines.

In **Figure** **6**, we show the distribution of the phonon lifetimes τ in Cs_3_Bi_2_I_6_Cl_3_ as a function of the phonon frequency. The Wigner limit (τ_*Wigner*_ = 1/Δω_*avg*_) is marked with horizontal dotted lines.^[^ ^38^ ^]^ Phonons with τ > τ_*Wigner*_ are mainly contributing to κ_*p*_. Δω_*avg*_ is average phonon interband spacing, $\Delta \omega_{avg}=\frac{\omega_{max}}{N}$ (ω_*max*_ being the maximum phonon frequency and *N* the number of phonon branches). The Ioffe–Regel limit (τ_*Ioffe* −*Regel*_ = 1/ω) is also presented with dotted lines. In all the compounds studied, there is a large population of phonons with τ_*Ioffe*−*Regel*_ < τ < τ_*Wigner*_, which is consistent with the results of a considerable κ_*c*_ component in κ_*L*_.

## 3 Conclusion 

In this work, we have demonstrated intrinsically ultralow κ_*L*_ in the halide perovskite Cs_3_Bi_2_I_6_Cl_3_ by the first‐principles phonon Boltzmann transport simulations. The dynamic rotation increases the aao scattering channels by softening the acoustic and low‐frequency optical phonons and introduces large lattice anharmonicity, leading to ultralow particle‐like thermal transport contributions. We reveal that the dynamic rotation is driven by the electrostatic repulsion of the *p*‐band electrons rather than the lone pairs formed by *s*‐band electrons. Moreover, due to the weak bonding of rattling Cs and Cl atoms with their surroundings, the optical phonon modes ≈1 and 2.2 THz exhibit flat and dense characteristics, which greatly contribute to glass‐like thermal transport. The origin of ultralow κ_*L*_ and large glass‐like contribution in Cs_3_Bi_2_I_6_Cl_3_ revealed by us are of great significance for understanding the thermal transport mechanism of halide perovskites.

## 4 Numerical Methods

The calculations are implemented using the Vienna Ab Initio simulation package (VASP) based on density functional theory (DFT)^[^ ^39^ ^]^ with the projector augmented wave (PAW) method and revised PBE‐GGA exchange–correlation functional for solids (PBEsol).^[^ ^40^ ^]^ The cutoff energy of the plane wave is set to 500 eV. The energy convergence value between two consecutive steps is set as 10^−5^ eV when optimizing atomic positions and the maximum Hellmann–Feynman (HF) force acting on each atom is 10^−3^ eV Å^−1^. The reciprocal space is sampled by a grid of 9 × 9 × 5 k points in the Brillouin zone. The calculations of κ_*L*_ and other relevant parameters such as phonon relaxation time are carried out by the ShengBTE software^[^ ^41^ ^]^ which operates based on the iterative scheme. The code used to calculate the glass‐like component in κ_*L*_ has been integrated into ShengBTE software by us.^[^ ^42^ ^]^ The **q**‐mesh in the first irreducible Brillouin Zone is set to be 10 × 10 × 10, the scale parameter for Gaussian smearing is set to be 0.1, and the supercells of 3 × 3 × 2 containing 252 atoms are chosen. The recently introduced on‐the‐fly Machine Learning Potential (FMLP) of VASP is used to accelerate ab initio molecular dynamics (AIMD) simulation, which has been verified to be reasonable for use in calculations of thermal transport properties.^[^ ^43^ ^]^ The simulation is run for 20 ps with a timestep of 1 fs. The second‐order, third‐order, and forth‐order IFCs are determined from the AIMD simulation by using the TDEP method^[^ ^44^ ^]^ with truncation radii 8, 5, and 3 Å, respectively and then converted to ShengBTE readable format. By using TDEP method, the renormalization of temperature effect on phonon frequency is taken into account. The thermal expansion is considered according to the quasiharmonic approximation (QHA) by Phonopy software.^[^ ^45^ ^]^

## Conflict of Interest

The authors declare no conflict of interest.

## Supporting information

Supporting Information

## Data Availability

The data that support the findings of this study are available from the corresponding author upon reasonable request.
