# Supplementary material for: Ultralow Lattice Thermal Conductivity and Large Glass‐Like Contribution in Cs3Bi2I6Cl3: Rattling Atoms and p‐Band Electrons Driven Dynamic Rotation
Source: Adv Sci (Weinh). 2024 Sep 18;11(42):2406380. doi: 10.1002/advs.202406380 (PMC11558099; doi:10.1002/advs.202406380)
Supplement: Supplementary file 1 — Supporting Information [file ADVS-11-2406380-s001.pdf]

ADVANCED SCIENCE
Open Access

Supporting Information

for *Adv. Sci.*, DOI 10.1002/advs.202406380

Ultralow Lattice Thermal Conductivity and Large Glass-Like Contribution in $Cs_3Bi_2I_6Cl_3$: Rattling Atoms and *p*-Band Electrons Driven Dynamic Rotation

Yu Wu, Jialin Ji*, Yimin Ding, Jiong Yang* and Liujiang Zhou**

Ultralow Lattice Thermal Conductivity and Large Glass-Like Contribution in $Cs_3Bi_2I_6Cl_3$: Rattling Atoms and p-Band Electrons Driven Dynamic Rotation (Supporting Information)

Yu Wu,[*,†] Jialin Ji,[*,‡] Yimin Ding,[†] Jiong Yang,[*,¶] and Liujiang Zhou[*,‖]

†*Yangtze Delta Region Institute (Huzhou), University of Electronic Science and Technology, Huzhou, Zhejiang 313001, China*

‡*College of Biological, Chemical Sciences and Engineering, Jiaxing University, Jiaxing, Zhejiang 314001, China*

¶*Materials Genome Institute, Shanghai University, Shanghai, 200444, China*

§*Zhejiang Laboratory, Hangzhou, Zhejiang, 311100, China*

‖*School of Physics and State Key Laboratory of Electronic Thin Films and Integrated Devices, University of Electronic Science and Technology, Sichuan, Chengdu 610054, China*

E-mail: wuyu9573@qq.com; jialinji@zjxu.edu.cn; jiongy@t.shu.edu.cn; ljzhou@uestc.edu.cn

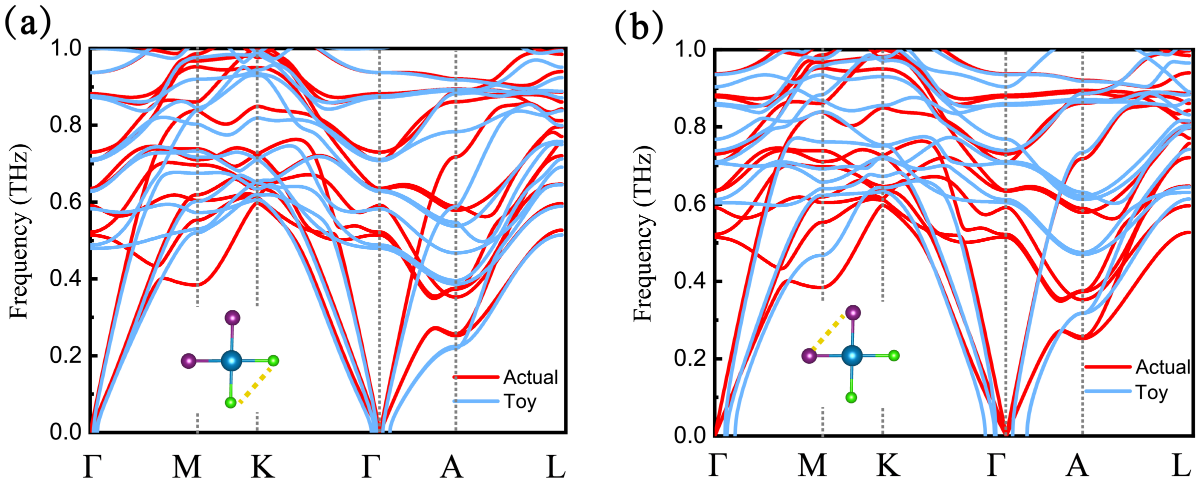

Figure S1: Actual phonon dispersion and toy model phonon dispersion by reducing the strength of the second-order IFCs of the neighboring (a) Cl-Cl and (b) I-I pairs in $Cs_3Bi_2I_6Cl_3$.

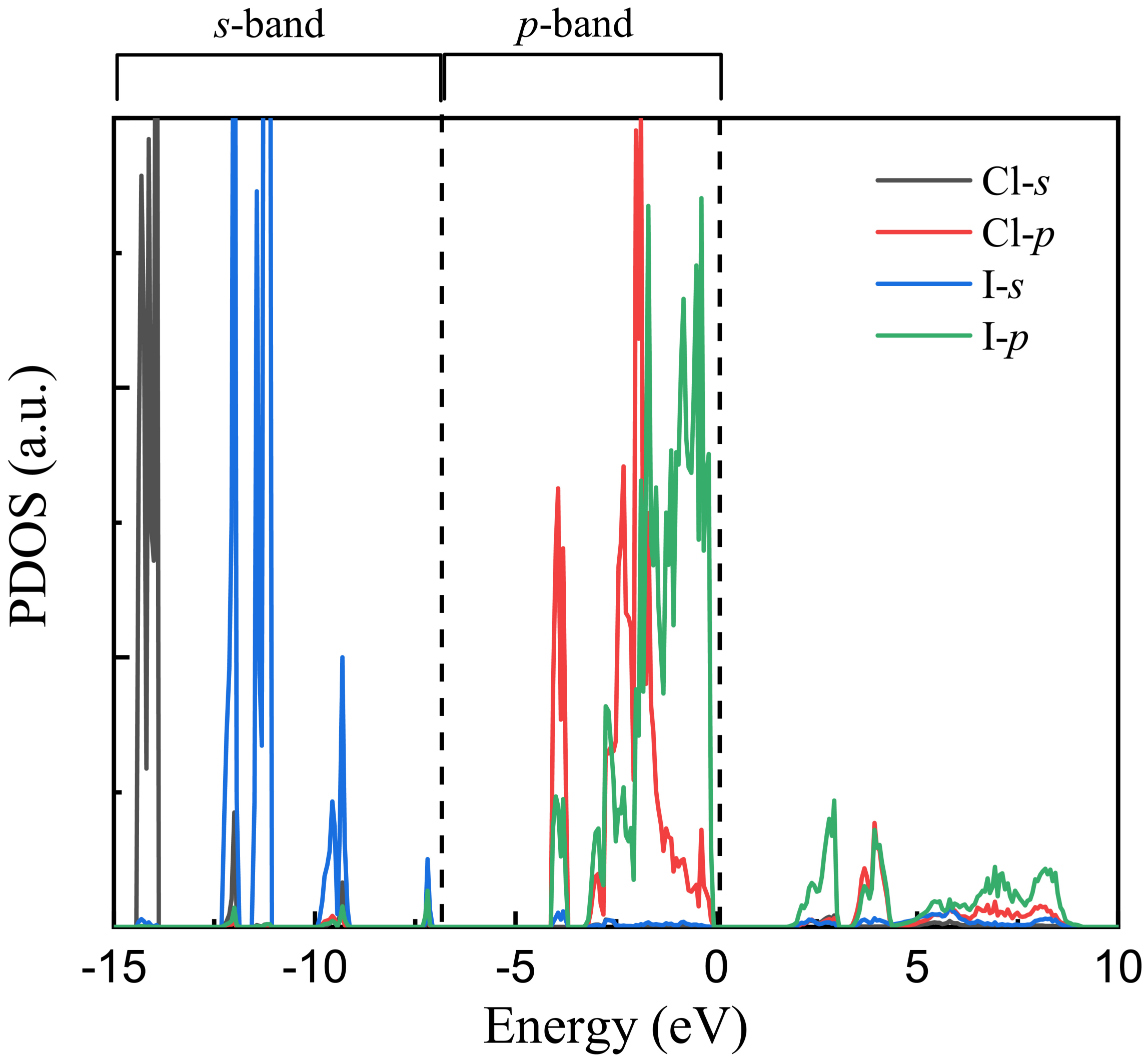

Figure S2: The PDOS of Cl and I atoms in $Cs_3Bi_2I_6Cl_3$.
